# NMDA Receptor Activity in Neuropsychiatric Disorders

**DOI:** 10.3389/fpsyt.2013.00052

**Published:** 2013-06-10

**Authors:** Shaheen E. Lakhan, Mario Caro, Norell Hadzimichalis

**Affiliations:** ^1^Biosciences Department, Global Neuroscience Initiative Foundation, Beverly Hills, CA, USA; ^2^Neurological Institute, Cleveland Clinic, Cleveland, OH, USA

**Keywords:** NMDA, psychiatry, schizophrenia, mood disorders, substance induced psychosis, Huntington’s disease, Alzheimer’s disease, neuropsychiatric systemic lupus erythematosus

## Abstract

*N*-Methyl-d-aspartate (NMDA) receptors play a variety of physiologic roles and their proper signaling is essential for cellular homeostasis. Any disruption in this pathway, leading to either enhanced or decreased activity, may result in the manifestation of neuropsychiatric pathologies such as schizophrenia, mood disorders, substance induced psychosis, Huntington’s disease, Alzheimer’s disease, and neuropsychiatric systemic lupus erythematosus. Here, we explore the notion that the overlap in activity of at least one biochemical pathway, the NMDA receptor pathway, may be the link to understanding the overlap in psychotic symptoms between diseases. This review intends to present a broad overview of those neuropsychiatric disorders for which alternations in NMDA receptor activity is prominent thus suggesting that continued direction of pharmaceutical intervention to this pathway may present a viable option for managing symptoms.

## Introduction

Diagnosis of psychiatric disorders is done clinically by focusing on observable symptoms and behaviors rather than on underlying psychodynamic processes or on the results of laboratory or imaging testing. Understanding the descriptive symptoms for mental disorders is vital in order to properly diagnose each psychiatric disease. The instruments most commonly used to diagnose and categorize mental illnesses, the Diagnostic and Statistical Manual of Mental Disorders (DSM-IV-TR) and the World Health Organization’s International Statistical Classification of diseases and Related Health Problems (ICD-10), focus on objective observations, without offering any discussion on the etiologies for these diseases. From a molecular, biochemical, and ultimately therapeutic perspective, it is equally as essential to characterize the role of various receptors, ligands, and neurotransmitters that when modified alter the manifestation of these symptoms.

Psychotic symptoms can be present in primary psychiatric disorders (schizophrenia, schizoaffective disorder, mood disorders, substance intoxication) and in psychiatric disorders that occur due to a medical condition such as Huntington’s disease (HD), Alzheimer’s disease (AD), and systemic lupus erythematosus (SLE). Several neurotransmitters have been linked to the development of psychotic symptoms, with dopamine and serotonin being the most widely studied due to the treatment effect of blocking certain subtypes of these receptors with antipsychotics. Unfortunately, long-term treatment with typical or atypical antipsychotics is limited due to side effects profile and high rates of discontinuation (Lieberman et al., 2005). *N*-Methyl-d-aspartate (NMDA) receptors have also been implicated in the development of psychotic symptoms and are a potential target for the development of novel treatments in the future.

### NMDA receptors in neuropsychiatric disorders

**Figure 1:**
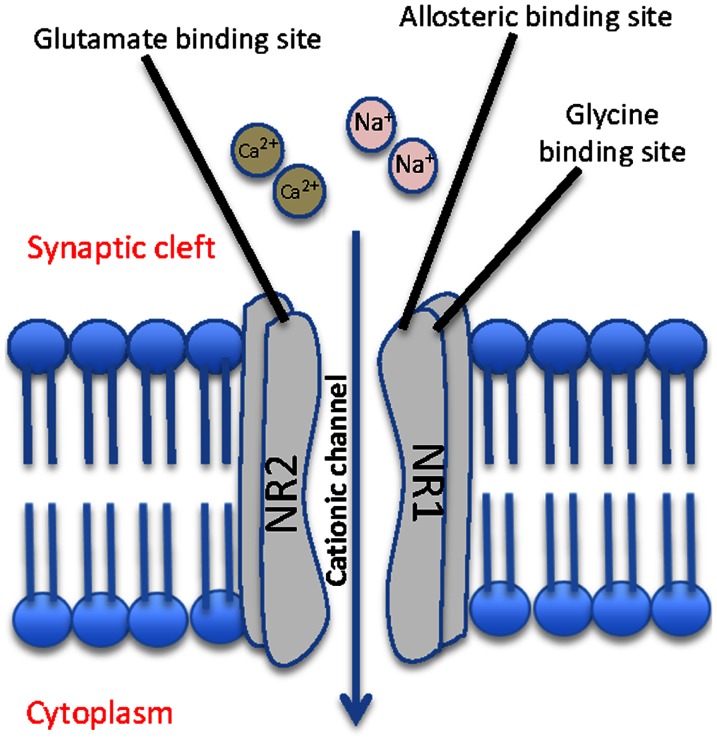
**Schematic representation of a typical NMDA receptor**. The NMDA contains four subunits, two glycine binding NR1 subunits and two glutamate binding NR2 subunits, and allows for cationic influx from the synaptic cleft into the cell.

*N*-Methyl-d-aspartate receptors are a class of glutamate receptor that when activated, mediate excitatory neurotransmission via passage of non-selective cations, including Ca^2+^, through the channel. They are abundantly and ubiquitously located throughout the brain and are understood to play a key role in synaptic plasticity and memory function (Stephenson et al., 2008; Li and Tsien, 2009). They are activated by binding the co-agonists glutamate and glycine, in addition to exposure to a positive change in membrane potential across the cell. Functional NMDA receptor heterotetramers are generally formed through a “dimer of dimers” mechanism and are conventionally made up of two glycine binding NR1 subunits and two glutamate binding NR2 subunits (Figure 1) (Dongen, 2009). While the NR1 subunit is considered essential to the formation of the complex, data indicates that the NR2 subunits may be interchangeable with either one or two NR3 subunits (Schuler et al., 2008).

The NMDA receptor is known to play an integral role in the regulation of signal transduction in multiple regions of the brain. Accordingly, any homeostatic dysfunction of NMDA receptor activity has the potential to result in a variety of pathologies. Previous authors have found a particularly high concentration of post-synaptic NMDA receptors in limbic structures (Kretschmer, 1999; Tsapakis and Travis, 2002), which is of uttermost importance in the pathogenesis of many psychiatric disorders.

Elucidation of the structural and mechanistic properties of NMDA receptors suggests a potential widespread use as a target for pharmacological intervention. Structurally, NMDA receptors provide a number of potentially viable sites for drug interaction. Initially, efforts focused on the development of broad-spectrum antagonists and ion channel blockers. However, more recent subtype selective targeting and strategic drug development aimed at these sites has resulted in the production of a variety of NMDA receptor agonists and antagonists that better address disease conditions ranging from mental disorders to pain management (Kemp and McKernan, 2002; Paoletti and Neyton, 2007).

*N*-Methyl-d-aspartate receptor antagonists describe a category of compounds that functionally inhibit or deactivate NMDA receptor activity. They can act broadly or specifically at various sites on the NMDA receptor including the agonist binding domains, allosteric sites, and the ion channel pore. This category of compounds has a potential use in any disease that results from glutamate-induced excitotoxicity ranging from cerebral ischemia and epilepsy to neurodegenerative disorders and neuropathic pain. More recent developments focus on subunit specific compounds, including NR2B-selective antagonists, and have resulted in minimization of side effects with improved therapeutic efficacy (Kemp and McKernan, 2002; Gogas, 2006; Paoletti and Neyton, 2007). NMDA receptor agonists, on the other hand, refer to the category of NMDA receptor targeted compounds that can enhance receptor activity. It is interesting to note, that some mental disorders can be treated with both NMDA receptor antagonists and agonists. These biphasic disorders in regards to NMDA receptor activity may require customized treatment protocols depending on the stage of the disease.

This review specifically focuses on neuropsychiatric disorders that manifest with psychotic symptoms and have the molecular commonality of NDMA receptor activity dysfunction. Here, we present a broad overview of the alterations in NMDA receptor activity in schizophrenia, bipolar disorder, HD, AD, substance induced psychosis, and neuropsychiatric systemic lupus erythematosus (NPSLE). We further emphasize the importance of continued pharmaceutical attention on this pathway as a target for the development of safer and more effective therapies.

## Schizophrenia

Schizophrenia is a psychotic disorder characterized by abnormalities in thought processing and content, presence of delusions and/or hallucinations as well as the presence of negative symptoms. Schizophrenia is a worldwide public health issue that affects approximately 1% of the adult population. While some symptoms and molecular pathways overlap with other related disorders including bipolar disorder, schizophrenia is unique in its precise presentation of positive, negative, and cognitive symptoms. For many years the *dopamine hypothesis* prevailed to explain symptoms associated with schizophrenia (Meltzer and Stahl, 1976). Thus, conventional antipsychotic drugs used to treat these patients would act by interfering with and inhibiting dopamine neurotransmission. While individuals using those drugs experienced significantly reduced positive psychotic symptoms, the effects were not optimal and often resulted in adverse side effects (Levinson, 1991; Fleischhacker, 1995). More recent elucidation of other neurotransmitter pathways involved in the manifestation of schizophrenia, such as serotonin and glutamate has provided novel treatment targets aimed at more effectively inhibiting both positive and negative symptoms (Fitzgerald et al., 1995; Iqbal and van Praag, 1995; Lindenmayer, 1995). Atypical antipsychotics act by blocking of serotonin type 2 and D_2_ receptors, initially, these medications were thought to be superior to typical antipsychotics due to less extrapyramidal side effects, however, recent evidence shows that there is also high discontinuation rate of atypical antipsychotics (Lieberman et al., 2005). Low treatment adherence has led investigators to study different and novel mechanisms for the development of novel medications for the management of psychosis.

Initial data in support of a more novel *glutamate hypofunction hypothesis* of schizophrenia arose from reports of low cerebrospinal fluid glutamate levels in patients with schizophrenia (Kim et al., 1980). Further studies corroborate this theory and indicate that administration of NMDA receptor antagonists including phencyclidine (PCP) and ketamine to patients with schizophrenia resulted in worsening of psychotic symptoms (Luby et al., 1959; Lahti et al., 1995; Gilmour et al., 2012). Additional studies reveal that administration of similar antagonists to healthy patients replicates symptoms of schizophrenia including positive, negative, and cognitive symptoms (Krystal et al., 1994; Gilmour et al., 2012).

Building on these data, more recent pharmacological approaches aimed at treating schizophrenia focus on the use of NMDA receptor agonists (Kemp and McKernan, 2002). However, direct activation of the receptor and reported excitotoxicity suggests the need to more specifically explore the glycine binding site as a potentially safer indirect target for treating glutamate hypofunction disorders (Lechner, 2006; Paoletti and Neyton, 2007). A number of studies are currently exploring this mechanism as a means of treating symptoms with minimal side effects. Both naturally occurring and synthetic glycine agonists including glycine, d-serine, and d-cycloserine are showing great promise for the treatment of positive and negative symptoms of schizophrenia (Coyle et al., 2003; Millan, 2005; Long et al., 2006). Following a similar mechanistic approach of indirectly targeting the glycine binding site, Glycine transport 1 (GLY-T_1_) inhibitors are being explored in order to modulate NMDA receptor function. The GLY-T_1_ reuptake pump functions to remove excess glycine in the synaptic cleft and thus inhibitors are being actively explored to increase glycine at the synapse. Animal data from transgenic mice suggest that the GLY-T_1_ inhibitor SSR103800 shows efficacy, decreased side effects, and suggests a use for GLY-T_1_ inhibitor as an adjunct to conventional therapy for schizophrenia (Boulay et al., 2010).

One of the largest trials performed so far studying the effect of increased glutamate transmission is the Cognitive and Negative Symptoms in Schizophrenia Trial (CONSIST) (Buchanan et al., 2007). The trial’s primary aim was to determine if co-administration of glycine (co-transmitter with glutamate at the NMDA receptor) or d-cycloserine (partial agonist at NMDA receptor) was associated with an improvement in cognitive impairment or in the negative symptoms of schizophrenia. During the trial, there was no improvement in the above mentioned symptoms with the experimental treatments. However, despite negative findings in this trial, there is clear evidence that NMDA receptor dysfunction is implicated in schizophrenia, and it is still an important research area for the development of future treatments.

Additional clinical evidence demonstrates that the GLY-T_1_ inhibitor Org 25935 has been explored for its antipsychotic properties. Preliminary human data indicate that it can effectively counteract the effects of the NMDA receptor antagonist, ketamine (D’Souza et al., 2012). Promising Phase II clinical data corroborate these results and further suggest that the GLY-T_1_ inhibitor RG1678 was a safe and effective compound for treating the negative symptoms of schizophrenia (Pinard et al., 2010).

The dopamine hypothesis and the glutamate hypofunction hypothesis of schizophrenia each separately explain specific aspects of the disease condition. However, some researchers argue that focusing on only one molecular pathway to characterize the complicated etiology of the disease is likely to narrow our understanding. In fact, some additional theories provide evidence that hypofunction of NMDA receptors results in dopaminergic abnormalities. Interestingly, this synergy between the two pathways best explains the positive, negative, and cognitive symptoms associated with the disease (Schwartz et al., 2012). Still, despite not agreeing on a molecular mechanism to explain the manifestation of schizophrenia, scientists do agree that NMDA receptor dysfunction plays an integral role and should continue to be studied as a therapeutic target.

## Mood Disorders

Mood is described as the internal feeling tone that influences the way an individual perceives himself and the environment. The most widely studied mood disorders are major depressive disorder, and bipolar affective disorder (BPAD), the latter one is characterized by alternation between manic and depressive episodes. Psychotic symptoms can be present in severe episodes of depression, mania, or during mixed states (Stahl, 2008). Mood disorders were initially thought to be caused by alterations in the levels of norepinephrine and serotonin, but this theory has not been able to completely explain the cause of this complex illness, this is evidenced by the results from a large-randomized clinical trial where only two-thirds of the patients who were treated with several treatment courses of different antidepressants experienced remission of their depression (Gaynes et al., 2008). Other theories include the alteration of hormonal regulation (hypothalamic-pituitary and thyroid axis dysfunction), alterations in γ-aminobutyric acid (GABA) receptors, and alterations in the arachidonic acid pathway and NMDA receptors.

The *arachidonic acid (AA) hypothesis* of BPAD represents a well-studied mechanism used to explain the pathophysiology of the disease. Support for this hypothesis gained popularity based on data showing that BPAD is associated with an upregulation in the AA cascade. These data were validated by reports that inhibitors of this cascade, including lithium, were successful mood stabilizers (Rapoport and Bosetti, 2002).

Similar to other mental disorders with psychotic manifestations, NMDA receptor activity seems to be intimately involved in the complicated web of pathways leading to the pathogenesis of mood disorders. The NMDA receptor antagonist, ketamine, has been shown to rapidly improve symptoms of depression (Zarate et al., 2006). Evidence from rat models has shown that the rapid improvement in depressive symptoms observed with ketamine is caused by an increase in the activation of the mammalian target of rapamycin (mTOR) pathway causing an increase in the number of neuronal synapses – opposite to changes seen during stress (Li et al., 2010). Other authors have described that ketamine also produces a disinhibition of GABAergic neurons, leading to increased presynaptic levels of glutamate; which then interacts with α-amino-3-hydroxy-5-methyl-4-isoxazolepropionic acid (AMPA) receptors, as the NMDA receptors are blocked by ketamine. This increased ratio of AMPA to NMDA activation and may be implicated in the antidepressant effects of ketamine (Sanacora et al., 2008; Andreasen et al., 2013).

Reports indicate there are elevated levels of glutamate in the left dorsolateral prefrontal cortex of adults with BPAD during the manic phase (Michael et al., 2003). More recent studies show that treatment with the NMDA receptor antagonist, MK-801, results in inhibition of the downstream AA pathway (Basselin et al., 2006). In addition, gene linkage analysis confirmed a role specifically for the NR2B subunit of the NMDA receptor in BPAD (Martucci et al., 2006). Together, these studies suggest that NMDA receptor activity plays a critical role in mood disorders likely through the in AA pathway.

## Huntington’s Disease

Huntington’s disease is a progressive genetic neurodegenerative disorder that results in decreased muscle coordination and cognitive ability. It is typically diagnosed between 30 and 40 years of age and often results in death within 15–20 years. In many cases, patients also present with psychiatric symptoms including some similar to those observed in patients with schizophrenia. On a molecular level, the underlying cause of HD is considered to be disruptions in the gene that encode the protein huntingtin, whose altered function ultimately leads to selective neuronal cell death (Gusella et al., 1983).

Some data suggest that the formation of nuclear protein aggregates plays a role in neuronal cell death associated with the disease (Carmichael et al., 2000). Others explore a variety of also likely contributing pathways including oxidative stress and impaired mitochondrial function (Davies and Ramsden, 2001). However, considering the focus of this review and the potential for manifestation of psychotic symptoms in HD, it is interesting to specifically note the role of the NMDA receptor pathway.

Early animal studies indicate that injections with kainic or quinolinic acids produce lesions similar to those observed in HD thus promoting the idea that NMDA receptor-mediated excitotoxicity may also contribute to the etiology of the disease (Coyle and Schwarcz, 1976; Beal et al., 1986). Related studies corroborate these early findings and report hyperactive NMDA receptors present in transgenic HD mice (Levine et al., 1999). Prolonged receptor activation results in excitotoxicity and cell death characteristic of many neurodegenerative disorders including HD. Further elucidation of this pathway explores a role for post-synaptic density protein 95 (PSD-95), a well-characterized scaffolding protein that binds to multiple cytoplasmic proteins including normal huntingtin and separately to the NR2 subunit of NMDA receptors. Once bound it causes clustering of receptors in the post-synaptic membrane and results in physiologic inhibition of NMDA receptor activity. In the case of HD, the presence of mutant huntingtin protein results in a disruption of PSD-95 binding to NMDA receptors, receptor hypersensitivity and resulting excitotoxicity, and ultimately increased neuronal cell death consistent with HD (Davies and Ramsden, 2001; Sun et al., 2001). Similar to schizophrenia and other complicated mental disorders, data point to the possibility of multiple and potentially parallel pathways giving rise to the variety of documented symptoms in HD. Nonetheless, the NMDA receptor pathway still remains a primary target for therapeutic intervention.

## Alzheimer’s Disease

Alzheimer’s disease describes a type of dementia that results in serious and progressive cognitive impairment. Symptoms range from commonly observed memory loss to psychotic manifestations including delusions. Currently, clinicians diagnose patients based on DSM-outlined criteria; however, a definitive diagnosis can only be made post-mortem.

For many years, the *amyloid hypothesis* of AD was the dominating model thought to explain the pathophysiology of the disorder. This hypothesis gained early popularity based on data that identified amyloid β-peptide (Aβ) as an integral component in the plaques observed post-mortem in AD (Masters et al., 1985). Additional reports confirm the central role of the Aβ protein and suggest that its accumulation initiates downstream diseases symptoms and molecular manifestations including alteration in the tau protein and the formation of characteristic neurofibrillary tangles (Hardy et al., 1998; Hardy and Selkoe, 2002).

As mentioned above, NMDA receptors are well-studied for their crucial role in learning and memory, key areas that are affected in the manifestation of AD. Similar to other mental disorders, AD is likely the result of dysfunction in multiple neurotransmitter pathways. In fact, data suggest an overlap in pathways related to NMDA receptor activation and production of characteristic biochemical and symptomatic changes that occur in AD. However, the exact nature of that overlap is not yet determined. Consistent with the idea that accumulation of Aβ protein initiates the pathological cascade, some studies suggest that the NMDA receptor may function indirectly as a receptor for the Aβ protein (Cisse et al., 2011; Malinow, 2012). A recent report from Cisse et al., indicates that the Aβ protein binds directly to the tyrosine kinase receptor, EphB2, a known regulator of NMDA receptor function (Cisse et al., 2011; Nolt et al., 2011). This results in degradation of EphB2 and a downstream reduction in NMDA receptor-mediated long term potentiation (Cisse et al., 2011). Other studies suggest that enhanced NMDA receptor activity results in increased processing of the amyloid beta precursor protein thus producing an increase in the AD characteristic Aβ protein. This in turn results in a decrease in excitatory synaptic transmission and may contribute to cognitive effects observed in early stages of AD (Gordon-Krajcer et al., 2002; Butterfield and Pocernich, 2003). With regards to the glutamatergic pathway, AD is unique in that receptor dysfunction is dependent on stage of the disease. In contrast with theories describing the pathogenesis of early stage AD, late stage pathogenesis is thought to be attributed to loss of NMDA receptors and resulting hypoactivity (Butterfield and Pocernich, 2003).

Precise mechanism aside, researchers agree that in conjunction with Aβ protein accumulation, the NMDA receptor pathway is integral in the manifestation of AD pathophysiology. Thus data point to the notion of a strategic combination therapy regimen to most effectively treat the condition.

## Substance Induced Psychosis

Substance induced psychosis (SIP) disorder represents another defined mental disorder that results in delusions or hallucinations that are linked to use or withdrawal from a variety of defined compounds. Interestingly, NMDA receptor antagonists PCP and ketamine, previously shown to produce an elevation of psychotic symptoms in patients with schizophrenia, are among the drugs reported to result in SIP (Luby et al., 1959; Lahti et al., 1995; Gilmour et al., 2012). Furthermore, there is evidence of potential for conversion from SIP to the more clearly defined psychotic disorder, schizophrenia (Rabe-Jablonska et al., 2012; Niemi-Pynttari et al., 2013). Although, the data in this space are mostly descriptive and the broad description of SIP gives rise to a poorly defined molecular mechanism, the likely role of NMDA receptors in the psychotic manifestation of this disease should not be left unnoted.

## Neuropsychiatric Systemic Lupus Erythematosus

Systemic lupus erythematosus is an autoimmune disorder that results in inflammation and threatens tissue damage to a number of different organ systems including the skin, heart, joints, lungs, and nervous system. Neuropsychiatric systemic lupus erythematosus is a poorly understood yet prevalent complication of SLE. It results when SLE specifically affects the central or peripheral nervous systems and patients manifest additional neurological symptoms such as psychosis, mood disorders, or cognitive dysfunction (Popescu and Kao, 2011). While the mechanism of NMDA receptor activity involvement is unique compared to the previously mentioned conditions, it is important to mention as it represents additional, albeit less clearly defined, evidence of an integral role for the NMDA receptor pathway in the pathogenesis of mental disorders.

Autoantibodies represent a type of antibody specifically targeted to endogenous proteins in an individual. They are understood to cause many of the symptoms associated with autoimmune disorders, including SLE. Previous data indicate that the cognitive manifestations of NPSLE may be related to medication and cardiovascular disease. However, additional data suggest that autoantibodies directed against various NMDA receptor subunits may also play a role in pathogenesis.

Studies show that NMDA receptor autoantibodies are prevalent in the manifestation of both mood symptoms and psychotic behaviors observed in patients with NPSLE (Mak et al., 2009). More specifically, autoantibodies to the NR2A and NR2B subunits of the NMDA receptor mediate neuronal death *in vitro* following breakdown of the blood brain barrier (Shefner et al., 1991; Katz et al., 1994; Gaynor et al., 1997; Kowal et al., 2004). Interestingly, the NMDA receptor antagonist memantine has been show to protect neurons from autoantibody-mediated cell injury, thus indicating that these autoantibodies may be acting as functional agonists (Lipton and Rosenberg, 1994; Kowal et al., 2004, 2006). Translating these results to human clinical trials, however, did not show similar efficacy (Petri et al., 2011). While these data may initially seem discouraging, there were some reported study limitations, including the use of physician confirmed, self-reported cognitive impairment. The authors indicate that memantine may still ultimately provide a therapeutic benefit in preventing the worsening of cognitive symptoms. Together, these data suggest that NMDA receptor antagonists represent a much underrated potential therapy for NPSLE.

## Conclusion

The role of the NMDA receptor pathway in a broad range of diseases and disorders is well established. However, how that “role” plays into the larger picture of disease manifestation is not entirely clear. Specifically, in the case of complicated mental disorders that manifest in a variety of symptoms including psychosis, the NMDA receptor pathway comes into play. Alterations in its activity lead to either hypofunction or hyperfunction and related excitotoxicity. To further complicate the matter, changes in NMDA receptor activity may be dependent on the stage of disease. For example, as indicated above, symptoms of AD can be initially attributed to glutamate-induced excitotoxicity. Late stage pathogenesis however, is thought to be a result, in part, of NMDA receptor hypoactivity.

Table 1 presents select NMDA receptor-targeted drugs that have been used as a monotherapy in clinical trials to alleviate symptoms of schizophrenia, BPAD, HD, AD, and NPSLE. Still, many other drugs and various drug combinations are also being explored in preclinical studies. While research has not yet clearly elucidated how these changes in receptor activity tie into the overlapping symptoms between mental disorders, we can be certain that maintaining receptor homeostasis is integral for symptom management. Combination therapy directed at selective NMDA receptor antagonists and enhancers in addition to drugs that target other disease-specific pathways may be the key to controlling the symptoms of psychotic disorders. However, more thorough molecular comparisons between the various mental disorders with psychotic manifestations is still needed.

**Table 1:** **Select NMDA receptor-targeted drugs used in clinical trials for the treatment of mental disorders**.

Drug	Primary disease target	Mechanism of action
D-Serine	Schizophrenia	NMDA receptor glycine site agonist
Sarcosine	Schizophrenia	NMDA receptor glycine site agonist
D-Amino acid oxidase inhibitor	Schizophrenia	NMDA enhancing agent via inhibition of the degradation d-serine levels
Clozapine	Schizophrenia	NMDA receptor enhancer
Memantine	Bipolar disorder	NMDA receptor antagonist
Memantine	Huntington’s disease	NMDA receptor antagonist
Amantadine	Huntington’s disease	NMDA receptor antagonist
Neramexane	Alzheimer’s disease	NMDA receptor antagonist
Memantine	Alzheimer’s disease	NMDA receptor antagonist
D-Amino acid oxidase inhibitor-B	Alzheimer’s disease	NMDA enhancing agent
Memantine	Neuropsychiatric systemic lupus erythematosus	NMDA receptor antagonist

This table presents select drugs that, at minimum, have been approved for initial clinical testing (NLM, 2013). This approval provides evidence of the important of the NMDA receptor pathway in the potential treatment of various mental disorders.

## Conflict of Interest Statement

The authors declare that the research was conducted in the absence of any commercial or financial relationships that could be construed as a potential conflict of interest.

## Abbreviations

AA, arachidonic acid; Aβ, amyloid beta; AD, Alzheimer’s disease; AMPA, α-amino-3-hydroxy-5-methyl-4-isoxazolepropionic acid; BPAD, bipolar affective disorder; CONSIST, Cognitive and Negative Symptoms in Schizophrenia Trial; DSM, Diagnostic and Statistical Manual of Mental Disorders; DSM-IV-TR, Diagnostic and Statistical Manual of Mental Disorders, fourth-edition, text-revision; GABA, in γ-aminobutyric acid; HD, Huntington’s disease; NMDA, *N*-Methyl-d-aspartate; SIP, substance induced psychosis; NPSLE, neuropsychiatric systemic lupus erythematosus; SLE, systemic lupus erythematosus.
